# Supplementary material for: Dissemination and stakeholder engagement practices among dissemination & implementation scientists: Results from an online survey
Source: PLoS One. 2019 Nov 13;14(11):e0216971. doi: 10.1371/journal.pone.0216971 (PMC6853327; doi:10.1371/journal.pone.0216971)
Supplement: S1 File — (DOCX) [file pone.0216971.s001.docx]

**Supporting Information: Survey of D&I Scientists**

We appreciate your participation in this important survey. Please try to answer each question, even if you are unsure. Your results will be used only in summary form so that it is not possible to identify your individual responses.

It takes most participants **between 10-15 minutes** to complete the entire survey.

Please note that once you begin, you must complete the survey in one setting as your answers will not be saved along the way.

For this survey, we define **dissemination** as:

An active and planned process that ensures that those who can use your research learn about it and can make use of the findings.

--------------------

1.. What methods do you usually use to disseminate research findings? (**Select all that apply**)

- Academic journals yes no
- Reports to funders yes no
- Press releases yes no
- Newsletters yes no
- Policy briefs Yes no
- Social media (Twitter, Instagram, Facebook, LinkedIn, etc) yes no
- Targeted mailings yes no
- Academic conferences yes no
- Other conferences yes no
- Seminars or workshops yes no
- Face-to-face meetings with stakeholders yes no
- Media interviews yes no
- Webinars or videos
- Other media yes no
- Other (please give details): ________________

2.. Of the methods you use to disseminate the research findings, which one do you think generally has the most impact on your career trajectory?

Choose one from those selected in Q1

3. Of the methods you use to disseminate the research findings, which one do you think generally has the most impact on practice or policy?

Choose one from those selected in Q1

***For the remainder of the survey,*** we ask that you focus specifically on the dissemination of research findings to non-research audiences such as healthcare providers, policymakers, community organizations, guideline organizations, patients, and/or other target populations.

4. To which of the following non-research audiences have you directly disseminated the findings of your research? Please select all that apply.

- Primary care clinics
- Hospitals or specialty care clinics
- VA or managed care organizations.
- State, provincial, or local health departments
- National/federal agencies
- Non-US governmental agencies
- Non-US non-governmental organizations
- Elected officials (e.g., Representatives; Parliament members; City council; State, provincial or regional officials)
- Non-profit organizations (e.g., American Cancer Society, Canadian Heart Association)
- Other community organizations or groups
- Health research funders
- The target population of your research (patients, communities)
- News media
- The general public
- Other (please specify)

5. Of the non-research audiences to which you disseminate research findings, which one do you think generally has the most impact on your career trajectory?

Choose one from those selected in Q4

6. Of the non-research audiences to which you disseminate research findings, which one do you think generally has the most impact on practice or policy?

Choose one from those selected in Q4

9. Is the dissemination of research findings to non-research audiences expected by your employer?

- Yes
- No
- Not sure

9a. [If yes]. How is your dissemination of research findings to non-research audiences evaluated (either by your employer or funding agencies)? 🡪 Open response

8. Which channels do you typically use to disseminate findings from your research to non-research audiences? (Select all that apply)

- I do not disseminate findings to non-research groups
- News media
- Policy briefs
- Social network activity
- One-on-one or small group meetings (with policymakers, key stakeholders, spokespeople, etc.)
- Workshops and seminars (marketed and designed for practitioners, policymakers, or other lay audiences)
- Webinars
- Other (Open response)

10. Do you think the dissemination of research findings to non-research audiences should be expected by your employer?

- Yes
- No
- Not sure

11. Do you think the dissemination of research findings to non-research audiences should be expected by your funding agencies?

- Yes
- No
- Not sure

12. How much do you agree with the following statement:

In my opinion, I receive enough credit professionally for my efforts disseminating research findings to non-research audiences?

(5 point Likert)

12a. (For those responding with 1 or 2 above) How would you improve the system for credit or recognition for disseminating research to non-research audiences?

(Open response)

13. How important to your own research is dissemination to non-research audiences?

- Very important
- Important
- Somewhat important
- Not important
- Not sure

14. How important to the work of your unit/department is dissemination to non-research audiences?

- Very important
- Important
- Somewhat important
- Not important
- Not sure

15. Is there a dedicated person or team responsible for dissemination-related activities within your unit/organization?

- Yes
- No
- Not sure

15a. (If yes) Is this person or team housed within a public relations/communication office?

- Yes
- No
- Not sure

16. What makes it difficult to disseminate your research findings to non-research audiences? Select all that apply

- Lack of understanding about how to disseminate findings beyond professional conferences and publications
- A low priority for research dissemination in my unit/department
- Uncertainty about what to disseminate
- Uncertainty about the impact of dissemination
- Lack of financial resources for dissemination
- Lack of staff time dedicated to dissemination
- Lack of organizational incentives for dissemination
- Unsure which organizations or groups want or would use the information
- Lack of relationships with stakeholders
- Dissemination activities not in study timelines
- Hesitation/resistance to disseminate findings from a single study
- Not sure results justify dissemination
- Other (please give details:

17. Over the past year, please estimate the proportion of your own time that is dedicated to dissemination-related activities to non-research audiences?

- None
- Less than 5% (*i.e.*, less than two hours a week)
- Between 5 and 25%
- Between 26 and 50%
- More than 50%

18. How often have you used a framework, theory, or logic model to plan dissemination-related activities?

- Always
- Usually
- Sometimes
- Rarely
- Never
- Not sure
- I do not plan dissemination-related activities.

18a. [If always, usually, sometimes, or rarely] Please list which frameworks or theories you most often use to plan dissemination-related activities:

19. When disseminating your program, policy, or procedure, how do you specify which elements should be implemented with fidelity?

- We present interventions as a package which needs to be implemented as designed
- We specify “core” elements which must be implemented with fidelity
- We specify both “core” elements, and ones which may be adapted/modified
- I do not plan dissemination-related activities.
- Other (please specify):

20. At what stage in the research process do you usually conduct dissemination-related activities? (Select all that apply)

- When the research is being formulated (at the proposal stage)
- During the implementation/data collection stage
- During the data analysis stage
- At the draft report/manuscript stage
- After results have been published or presented at research meetings
- I do not plan dissemination activities

21. When engaging non-research stakeholders (e.g., patients, community members, practitioners, policymakers) in your research process, how many times do you meet with these stakeholders across a project?

- Once
- Two or three times
- Four or more times
- I don’t typically engage non-research stakeholders during the research process

21a. [For those who answered Once, twice, or three or more] How do you involve these **stakeholder groups** in your research and dissemination efforts? Please check all that apply

|  | **Focus Groups** | **Advisory Committees** | **User Panels (“beta” testing)** | **Data Gathering** | **Provide Testimonials or Endorsements** | **Formal Research Team Members** | **Interpreting Data** | **Writing Up Findings** | **Disseminating Findings Through Personal and Professional Networks** |
| --- | --- | --- | --- | --- | --- | --- | --- | --- | --- |
| **Patients/Consumers** |  |  |  |  |  |  |  |  |  |
| **Direct Practitioners** |  |  |  |  |  |  |  |  |  |
| **Organizational Decision Makers** |  |  |  |  |  |  |  |  |  |
| **Policymakers** |  |  |  |  |  |  |  |  |  |

Other (please give details): ____________________

22. How often do you produce research summaries or key messages that are written for specific non-research audiences or groups (such as key community groups or practice organizations)?

- Always
- Usually
- Sometimes
- Rarely
- Never

23. How often do you evaluate the uptake of your research in new settings, changing health practice or policy (e.g. use of your research by a health department, use of your research for the basis for training of practitioners)?

- Always
- Usually
- Sometimes
- Rarely
- Never

23a. **[If yes] What method(s) do you use to evaluate the uptake of your research in new settings? Please select all that apply.**

- Surveys
- Qualitative methods
- Other: ________

24. Please indicate your level of agreement with the following statement:

It is an obligation of researchers to disseminate their research (translate knowledge) to those who need to learn about it and make use of the findings.

- Strongly agree
- Agree
- Neither agree or disagree
- Disagree
- Strongly disagree

26. Where do you work?

- University or research organization (US-based)
- University or research organization (Canadian-based)
- University or research organization (other country-based)
- NIH or CIHR
- Center for Disease Control, or national/regional public health organization
- WHO
- USAID
- Private Nonprofit Organization
- Private For Profit Organization
- Other, please specify:________________

27. In what setting do you do **most** of your research (choose one)?

- Clinical (inpatient or outpatient)
- Community settings
- Health departments
- Health delivery systems
- Policy settings
- My own academic setting
- Other (please specify): ______

27. Please check all of the following that apply:

- I am affiliated with the CDC’s Prevention Research Centers Program.
- I have received NIH or CIHR funding for dissemination and implementation research.
- I have received PCORI funding for dissemination and implementation or large pragmatic trials research.
- I have received other funding for dissemination and implementation research, please specify:________________

28. What are the academic areas of your formal graduate degrees and/or fellowships? Please select all that apply.

- Behavioural Science (e.g. Social Work, Psychology)
- Natural Sciences (e.g. Biology, Chemistry, Physics)
- Medicine
- Nursing
- Public Health (including Epidemiology)
- Policy
- Health Services Research
- Other (please specify):___________________

29. In what year did you receive your highest academic degree? _____

Have you received formal post-doctoral training in knowledge translation or dissemination and implementation research?

- Yes
- No

(If yes) What format did this training take?

- Fellowship or other long-term training
- University course (at least a quarter or semester in length)
- Multi-day short course
- Online course
- Single day presentation

31. Have you ever worked in a practice or policy setting where your research might be applicable?

- Yes
- No
- Not sure

25. You may use this space to leave any questions or comments about this survey. Again, your responses are anonymous.

By clicking the forward arrow, you will SUBMIT your responses.

Thank you for taking the time to complete the questionnaire!
